# Association of SARS-CoV-2 infection with physical activity domains and types

**DOI:** 10.1038/s41598-023-46162-4

**Published:** 2023-11-06

**Authors:** Jérémy Vanhelst, Bernard Srour, Laurent Bourhis, Hélène Charreire, Charlotte Mélanie VerdotDeschasaux-Tanguy, Nathalie Druesne-Pecollo, Fabien Szabo de Edelenyi, Julien Allègre, Benjamin Allès, Valérie Deschamps, Alice Bellicha, Leopold K. Fezeu, Pilar Galan, Chantal Julia, Emmanuelle Kesse-Guyot, Serge Hercberg, Nathalie Bajos, Gianluca Severi, Marie Zins, Xavier de Lamballerie, Fabrice Carrat, Jean-Michel Oppert, Mathilde Touvier, the SAPRIS, SAPRIS-SERO Study Groups, Hélène Blanché, Jean-François Deleuze, Clovis Lusivika-Nzinga, Gregory Pannetier, Nathanael Lapidus, Isabelle Goderel, Céline Dorival, Jérôme Nicol, Olivier Robineau, Sofiane Kab, Adeline Renuy, Stéphane Le-Got, Céline Ribet, Mireille Pellicer, Emmanuel Wiernik, Marcel Goldberg, Fanny Artaud, Pascale Gerbouin-Rérolle, Mélody Enguix, Camille Laplanche, Roselyn Gomes-Rima, Lyan Hoang, Emmanuelle Correia, Alpha Amadou Barry, Nadège Senina, Younes Esseddik, Mélanie Deschasaux, Jean-Marc Sébaoun, Jean-Christophe Beaudoin, Laetitia Gressin, Valérie Morel, Ouissam Ouili, Laetitia Ninove, Stéphane Priet, Paola Mariela Saba Villarroel, Toscane Fourié, Souand Mohamed Ali, Abdenour Amroun, Morgan Seston, Nazli Ayhan, Boris Pastorino

**Affiliations:** 1 Université Sorbonne Paris Nord and Université Paris Cité, INSERM, INRAE, CNAM, Center of Research in Epidemiology and StatisticS (CRESS), Nutritional Epidemiology Research Team (EREN), 93017 Bobigny, France; 2 MoISA, Univ Montpellier, CIRAD, CIHEAM-IAMM, INRAE, Institut Agro, IRD, Montpellier, France; 3 Santé Publique France, Nutritional Surveillance and Epidemiology Team (ESEN), Sorbonne Paris Nord University, Epidemiology and Statistics Research Center–University of Paris (CRESS), Bobigny, France; 4 Département de Santé Publique, APHP, Sorbonne Université, Paris, France; 5 IRIS, UMR CNRS 8156, EHESS, Inserm U997, Aubervilliers, France; 6 Paris-Saclay University, UVSQ, Inserm, Gustave Roussy, “Exposome and Heredity” Team, CESP UMR1018, Villejuif, France; 7 Department of Statistics, Computer Science and Applications “G. Parenti”, University of Florence, Florence, Italy; 8 Paris University, Paris, France; 9 Inserm UMS 11, Paris Saclay University, Villejuif, France; 10 Unité des Virus Emergents (UVE), Aix Marseille Univ, IRD 190, INSERM 1207, Marseille, France; 11 Sorbonne Université, Inserm, Institut Pierre-Louis d’Epidémiologie et de Santé Publique, Paris, France; 12 Department of Nutrition, Human Nutrition Research Center Ile-de-France (CRNH IdF), Pitié-Salpêtrière Hospital (AP-HP), Sorbonne University, Paris, France; 13 Fondation Jean Dausset-CEPH (Centre d’Etude du Polymorphisme Humain), CEPH-Biobank, Paris, France; 14 Institut Pierre-Louis d’Épidémiologie et de Santé Publique, Sorbonne Université, Inserm, Paris, France

**Keywords:** Diseases, Risk factors

## Abstract

Lockdown imposed in the early phase of the SARS-CoV-2 outbreak represented a specific setting where activity was restricted but still possible. The aim was to investigate the cross-sectional associations between physical activity (PA) and SARS-CoV-2 infection in a French population-based cohort. Participants completed a PA questionnaire. PA was classified into: (*i*) total PA; (*ii*) aerobic PA by intensity; (*iii*) strengthening PA; (*iv*) PA by domain and type; and (*vii*) by location. Sedentary time was also recorded. Seroprevalence of anti-SARS-CoV-2 antibodies was assessed. Multivariable logistic regression models controlling for sociodemographic, lifestyle, anthropometric data, health status, and adherence to recommended protective anti-SARS-CoV-2 behaviours were computed. From 22,165 participants included, 21,074 (95.1%) and 1091 (4.9%) had a negative and positive ELISA-S test result, respectively. Total PA, vigorous PA, leisure-time PA, household PA, outdoor PA and indoor PA were all associated with lower probability of SARS-CoV-2 infection. Observations made in such a setting shed light on PA possibilities in a context of restricted mobility, where the health benefits of PA should not be overlooked. Along with already well-established benefits of PA for non-communicable disease prevention, these findings provide additional evidence for policies promoting all types of PA as a lever for population health.

## Introduction

Following the worldwide pandemic caused by the (Severe acute respiratory syndrome associated coronavirus-2 (SARS-CoV-2) virus, there has been great interest in identifying risk or protective factors for the resulting coronavirus disease (COVID-19) and related health consequences. Improving our understanding of factors associated with COVID-19-related outcomes is much needed for designing present and future global and national health prevention policies. Through a number of systematic reviews and meta-analyses, there is strong evidence of links between individual characteristics (e.g. older age, sex (males), heavy current smoking, presence of chronic disease such as obesity, diabetes, hypertension, cardiovascular and respiratory diseases, or cancer) and increased risk of SARS-CoV-2 infection, morbidity, severity and related death^1–5^. There is currently less evidence regarding the protective role of lifestyle factors including physical activity (PA).

Beyond the established health-enhancing effects of regular PA regarding non-communicable diseases (NCDs) and associated risk factors^6^, a protective role of regular PA is well documented against infectivity and severity of respiratory infections^7,8^. A systematic review and meta‑analysis concluded that regularly engaging in moderate to vigorous PA (MVPA) is associated with a 31% risk reduction of community-acquired infectious disease and 37% risk reduction in infectious disease mortality^9^. It is therefore highly relevant to delineate the role of PA towards COVID-19 risk. Even if one study on a large sample showed a protective effect of PA regarding severe COVID-19 illness and mortality, the body of knowledge in this field rely on a still relatively limited number of studies^10–14^.

Some limitations of previous work on relations between PA and COVID-19 risk were outlined in a recent systematic review and meta-analysis^15^. First, studies only used data on leisure-time PA and did not address the potential contribution of other important PA domains such as occupation- or household-related PA. Then, authors stressed the fact that no study accounted for factors such as social distancing, mask wearing and hand washing, while individual hygiene and the respect of protective barrier behaviours may seriously confound studied associations with risk of SARS-CoV-2 infection. In addition, drastic measures (permitting only 1 h per day recreational activity in a 1 km radius around residential address) imposed during lockdown resulted in an unprecedented disruption of daily life, leading to an unplanned natural experiment that may be used to understand the effects of an enforced limitation of movement on health. To address these limitations, we decided to perform a cross-sectional study in a large French cohort assessing the relationships between PA, including detailed information on multiple domains of PA and types, assessed during the first French lockdown (April–May 2020), and the SARS-CoV-2 infection obtained by seroprevalence from March 2020 to following 6 months, taking into account protective barrier behaviours.

Therefore, the aim of this study was to investigate the relationship between PA domains and types during the lockdown and the SARS-CoV-2 infection in a large French population-based sample.

## Methods

### Study population: the NutriNet-Santé cohort

The current report is based on data collected from the multi-cohort SAPRIS (“SAnté, Perception, pratiques, Relations et Inégalités Sociales en population générale pendant la crise COVID-19”) survey involving the NutriNet-Santé study. The SAPRIS survey started at the beginning of the first French lockdown, in April 2020 to assess the main epidemiological, social and behavioural challenges of the COVID-19 epidemic in France in relation to social inequalities in health and healthcare. Data were collected between April 1st, 2020 and May 12th, 2020. It was approved by the INSERM (French National Institute for Health and Medical Research) Ethics Evaluation Committee (approval #20–672 dated March 30th, 2020).

The NutriNet-Santé study is an ongoing web-based cohort launched in France in May 2009. The aim of this web-based cohort is to assess the relationships between nutrition and health along with the determinants of nutrition-related behaviours. A detailed description has been published elsewhere^16^. Volunteers aged 15 years or older living in France and having access to the Internet fill in self-administered web-based questionnaires at baseline as well as at regular time points during follow-up using a dedicated website. The NutriNet-Santé study is conducted in accordance with the Declaration of Helsinki, and all procedures were approved by the Institutional Review Board of the French Institute for Health and Medical Research (IRB INSERM #0000388FWA00005831) and by the National Commission on Informatics and Liberty (CNIL #908,450 and #909,216). All participants provided informed consent and an electronic signature. The study is registered at ClinicalTrials.gov (NCT03335644).

### Measurements

#### Physical activity

During the first lockdown (March 17th, 2020–May 3rd, 2020), a set of ad hoc questionnaires was sent to the NutriNet-Santé participants to collect extensive data on health status and lifestyle, including PA at the beginning of lockdown^17^. Participants were asked to report time spent in various types of PA during the past 7 days, as previously described^17^. Participants were asked to fill in the PA questionnaire at the same time that they completed the SAPRIS questionnaire, between April and May 2020. Activities surveyed included: brisk walking, walking a pet, jogging, cycling (outdoor and indoor), treadmill walking/running, rowing, aerobics, dancing, strength training, yoga, stretching, active play with children, household chores (cleaning), gardening and craft activities, and other relevant activity. PA was classified into several categories: (*i*) total PA; (*ii*) aerobic PA by intensity (moderate, vigorous); (*iii*) strengthening PA; (*iv*) PA by type (leisure-time, household, total walking); and (*vii*) by location (outdoor, indoor). Time spent was collected for each PA domain and expressed as min/week. In addition, we classified PA according to metabolic equivalent task (MET) score to take into account PA intensity. A MET is the ratio of the working metabolic rate of an activity divided by the resting metabolic rate. One MET represents the metabolic rate of an individual at rest (sitting quietly) and is set at 3.5 ml of oxygen consumed per kg body mass per minute, or approximately 1 kcal/kg/h^18^. PA of light, moderate, and vigorous intensities were rated as > 1.5, > 3, and > 7 METs, respectively. We calculated the MET-minutes per week by multiplying the standard MET score by PA duration and frequency per week^18^. Sedentary time (min/day), including screen time and sedentary behaviour (any waking behaviour characterised by an energy expenditure of 1.5 METs or lower while sitting, reclining or lying) was also recorded.

#### SARS-CoV-2 seroprevalence

To determine whether participants have been infected by SARS-CoV-2, we invited participants who completed the COVID-19-related SAPRIS questionnaires to take part in the SAPRIS-SERO project (approved by CPP Sud-Méditerranée III on April 27, 2020, and CNIL #920193, electronic informed consent was obtained from all participants for dried-blood spot testing)^19^. Volunteer participants received self-sampling dried-blood spot kits by mail between May and October 2020. After processing, serological analyses were performed using commercial Enzyme-linked immunosorbent assay (ELISA) tests (Euroimmun^®^, Lübeck, Germany) to detect anti-SARS-CoV-2 antibodies (immunoglobulin G, IgG) directed against the spike protein S1 domain (ELISA-S). The ELISA-S test was considered positive for values of optical density ratio ≥ 1.1, indeterminate for values between 0.8 and 1.1, and negative for values < 0.8. The main outcome was a positive ELISA-S test. Participants with ELISA-S results in the indeterminate range were excluded from the analyses.

#### Covariates

NutriNet-Santé participants regularly complete web-based questionnaires. Upon inclusion and then every year or 6 months, a set of five questionnaires related to sociodemographic and lifestyle characteristics (sex, educational level, smoking status, alcohol intake, marital status), anthropometric data (height and weight), health status (personal and family history of diseases, drug treatment), dietary intakes (using three non-consecutive web-based 24-h dietary records randomly assigned over a two-week period including two weekdays and one weekend day) and PA (using the International Physical Activity Questionnaire—IPAQ) are sent to the participants^20–22^. For the present analysis, we used the last questionnaires completed by participants before the lockdown, except for energy intake. Food consumption and nutrient intakes were calculated as an average per day, 24-h dietary records available (minimum: 6) from January 1st, 2018, to February 1st, 2020, i.e., the 2 years preceding the start of the COVID-19 pandemic in France.

In addition, a specific COVID-19 research protocol was set up in April 2020 as part of the SAPRIS nationwide multi-cohort project, including several questionnaires repeatedly collecting information about participants’ SARS-CoV-2 infection/diagnosis and experience of lockdown (e.g., employment status during lockdown, presence of children and or grandchildren at home, frequency of going out in the past week, body weight before the lockdown)^19^. From these questionnaires, a composite score reflecting the adherence to recommended protective behaviours against SARS-CoV-2 infection was computed^23^. The index was calculated as the average sum of points attributed to hand washing when going back home (always: 3, almost always: 2, sometimes: 1, never: 0), mask-wearing (always: 3, sometimes: 1.5, never: 0), and physical distancing (> 1 m from others: 3, > 1 m from almost everybody:1.5, < 1 m: 0) and ranged from 0 to 9. All NutriNet-Santé questionnaires are available online (in French) at https://info.etude-nutrinet-sante.fr.

#### Statistical analysis

**Figure 1:**
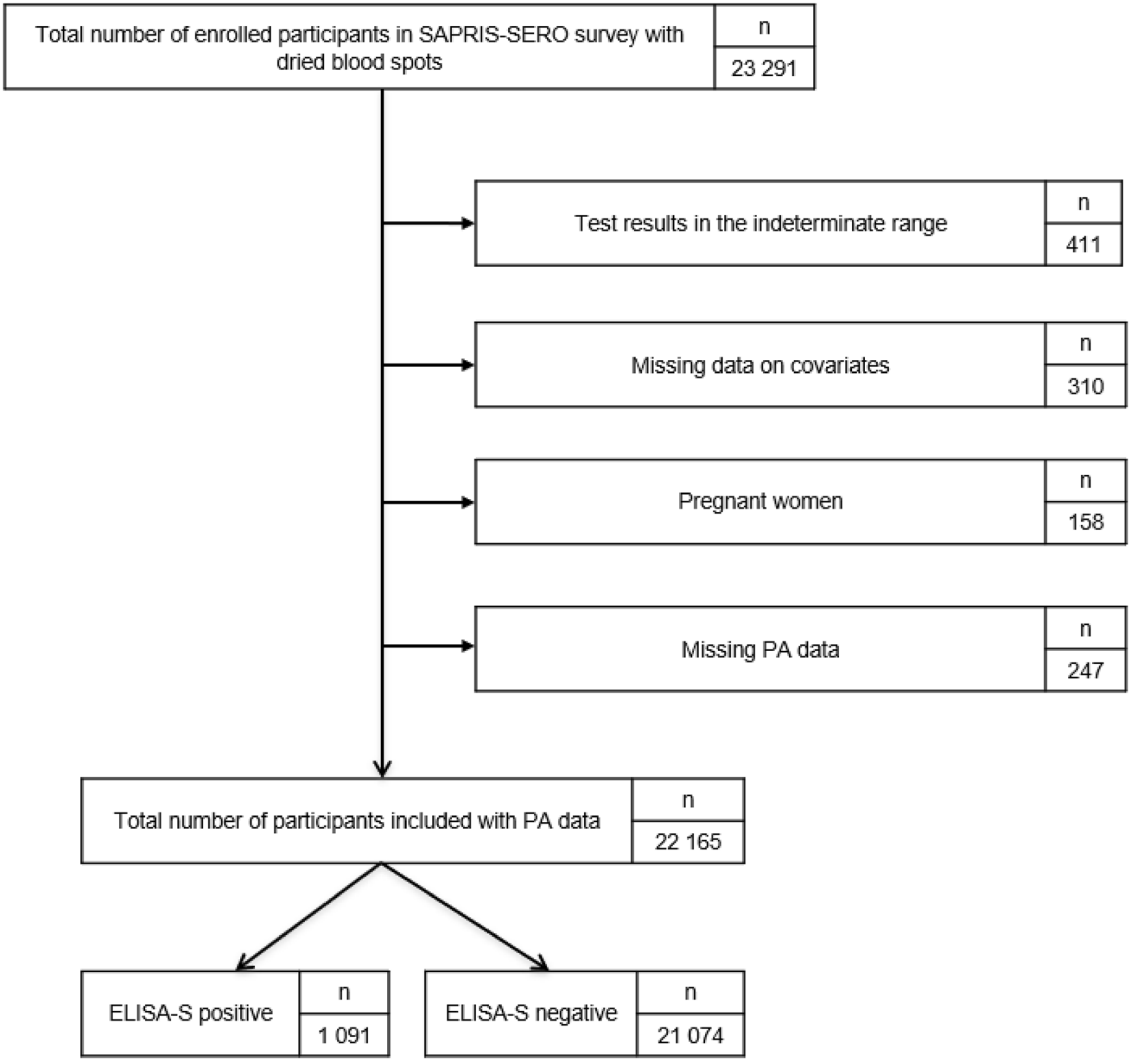
Participants flowchart, NutriNet-Santé cohort (2009–2020)—SAPRIS-SERO, France.

From 26,002 patients with a dried blood spot, 22,165 participants completed the PA questionnaire at the beginning of lockdown with either a positive or a negative ELISA-S test and were thus included in the analyses (Flowchart presented in Fig. 1).

Associations between PA domains and types at the beginning of lockdown and the probability of SARS-CoV-2 infection (positive vs. negative ELISA-S test) were assessed using multivariable logistic regression models. According to previous studies on dose–response associations between PA and sedentary time and mortality, specific increments were defined for PA duration and sedentary time^24,25^. PA duration for each domain and type and sedentary time were handled as continuous variables and the odds ratios (OR) and 95% confidence intervals (CI) were computed for an increment of 30-min per week. Sedentary time were also handled as continuous variables and the odds ratios (OR) and 95% confidence intervals (CI) were computed for an increment of 420-min per week. Additionally, only for PA duration, the OR (95% CI) were computed for an increment of 250 MET-minutes per week. Models were adjusted for the following characteristics, assessed in April 2020 (i.e., during the first lockdown/ wave of the epidemics in France): sex, age, educational level, employment status, smoking status, presence of children and/or grandchildren aged under 18 years at home, residential area, geographical area, frequency of going out over the past week and prevalent chronic disease (cancer, cardiovascular disease, high blood pressure, diabetes, dyslipidemia), close relatives with COVID-19 symptoms, body mass index (BMI) and the month of blood draw. Models also included a composite index reflecting the adherence to 3 recommended protective behaviours when going out, assessed twice (questionnaires in April and May 2020).

In sensitivity analyses on a subsample with available data, energy intake (without alcohol) and daily PA levels, assessed by IPAQ, before the lockdown were included into the models. We also tested restricting our study sample to a nested case–control design, with matching for age, sex, and residential area (4 controls per case).

All tests were two-sided and *P* < 0.05 was considered statistically significant. Analyses were carried out using SAS 9.4 (SAS Institute Inc., USA).

### Ethical approval and consent to participate

The NutriNet-Santé study is conducted in accordance with the Declaration of Helsinki, and all procedures were approved by the Institutional Review Board of the French Institute for Health and Medical Research (IRB INSERM #0000388FWA00005831) and by the National Commission on Informatics and Liberty (CNIL #908,450 and #909,216). All participants provided informed consent and an electronic signature. The study is registered at ClinicalTrials. gov (#NCT03335644). The SAPRIS protocol was approved by the Inserm ethics committee (approval #20-672 dated March 30, 2020). The SAPRIS-SERO study was approved by the Sud-Mediterranée III ethics committee (approval #20.04.22.74247) and electronic informed consent was obtained from all participants for dried-blood spot testing.

## Results

### Description of the study sample

Characteristics of included participants according to ELISA-S tests status are described in Table 1. From 22,165 participants included, 21,074 (95.1%) and 1,091 (4.9%) had a negative and positive ELISA-S tests results, respectively. ELISA-S positive participants were younger and more often women, more likely to have a graduate degree, to have a professional activity (outside home and from home) during the lockdown (March–May 2020), to have children aged under 18 years at home, to have a lower pre-lockdown PA level, to be non-smokers, to live in large cities, to live in Paris city or inner suburbs or in the eastern part of France, and were more likely to have a prevalent chronic disease. ELISA-S positive participants were also more likely to have close relatives with COVID-19 symptoms at the time of blood spots.

**Table 1:** Characteristics of participants included according to ELISA-S tests status, NutriNet-Santé cohort study, April 2020—SAPRIS-SERO, France.

	ELISA-S negative (n = 21,074) n (%) mean ± SD	ELISA-S positive (n = 1091) n (%) mean ± SD	P*
Sex			< 0.001
Men	5272 (25.0)	223 (20.4)	
Women	15,802 (75.0)	868 (79.6)	
Age (*years*)	57.3 ± 13.6	49.5 ± 13.2	< 0.0001
Educational level			< 0.0001
< High school degree	3204 (15.2)	86 (7.9)	
High schhol degree	2563 (12.2)	97 (8.9)	
Undergraduate degree	6706 (31.8)	313 (28.7)	
Graduate degree	8601 (40.8)	595 (54.5)	
Professional activity			< 0.0001
Unemployment	10,735 (50.9)	300 (27.5)	
Short time working	2104 (10.0)	166 (15.2)	
Working outside home	1830 (8.7)	111 (10.2)	
Working from home	5785 (27.4)	478 (43.8)	
Student	620 (3.0)	36 (3.3)	
Residential area			< 0.0001
Rural area	7327 (34.8)	294 (27.0)	
City < 20,000 inhabitants	5040 (23.9)	261 (23.9)	
City ≥ 20,000 to < 100,000 inhabitants	4687 (22.2)	286 (26.2)	
City ≥ 100,000 inhabitants	4020 (19.1)	250 (22.9)	
Region			< 0.0001
Paris basin	2966 (14.1)	116 (10.6)	
Centre-East	3118 (14.8)	140 (12.8)	
East	1686 (8.0)	157 (14.4)	
Mediterranean	2685 (12.7)	66 (6.1)	
North	732 (3.5)	35 (3.2)	
West	3275 (15.5)	101 (9.3)	
Paris and closed suburb	4048 (19.2)	394 (36.1)	
Southwest	2564 (12.2)	82 (7.5)	
Number of persons in the household	2.4 ± 1.3	2.7 ± 1.5	< 0.0001
Presence of children/grandchildren aged under 18 years at home			< 0.0001
Yes	4624 (21.9)	444 (40.7)	
No	16,450 (78.1)	647 (59.3)	
Close relatives with COVID-19 symptoms at the time of blood spots			< 0.0001
Yes	9508 (45.1)	714 (65.4)	
No	11,566 (54.9)	377 (34.6)	
BMI (kg/m^2^)	24.2 ± 4.5	24.2 ± 4.7	0.91
Smoking status			< 0.0001
Non-smoker	8914 (42.3)	563 (51.6)	
Former smoker	10,671 (50.6)	459 (42.1)	
Current Smoker	1489 (7.1)	69 (6.3)	
Chronic disease			< 0.0001
Yes	6970 (33.1)	248 (22.7)	
No	14,104 (66.9)	843 (77.3)	
Protective behavior index	5.9 ± 1.4	5.9 ± 1.5	0.28
Frequency of going out over the past week			< 0.0001
Never	1632 (7.7)	119 (10.9)	
Once	4696 (22.3)	253 (23.2)	
2 to 5 times	8898 (42.2)	470 (43.1)	
6 to 10 times	4609 (21.9)	206 (18.9)	
More to 10 times	1239 (5.9)	43 (3.9)	
PA level pre-lockdown (IPAQ)			< 0.0001
Low	3077 (14.6)	170 (15.6)	
Moderate	7815 (37.1)	466 (42.7)	
High	8995 (42.7)	363 (33.3)	

*P values from chi-square tests (categorical variables) or Fisher tests (quantitative variables) for unadjusted associations between individual characteristics and ELISA-S test status.

### PA domains and types with ELISA-S status

PA domains and types according to ELISA-S status are shown in Table 2. In these unadjusted analyses, we observed that ELISA-S positive participants spent significantly less time in PA compared to ELISA-S negative participants. In contrast, ELISA-S positive participants spent significantly more time in sedentary behaviours compared to ELISA-S negative participants.

**Table 2:** Physical activity of included participants according to ELISA-S tests status, NutriNet-Santé cohort study, April 2020—SAPRIS-SERO, France.

	Overall	ELISA-S negative	ELISA-S positive	P*
Total PA				
Min/week	680 ± 594	690 ± 596	498 ± 522	< 0.0001
MET-minutes/week	2803 ± 2465	2843 ± 2474	2033 ± 2149	< 0.0001
Moderate PA				
Min/week	155 ± 205	157 ± 206	118 ± 184	< 0.0001
MET-minutes/week	541 ± 700	548 ± 702	416 ± 632	< 0.0001
Vigorous PA				
Min/week	56 ± 102	57 ± 103	46 ± 86	0.09
MET-minutes/week	386 ± 699	390 ± 704	315 ± 591	0.09
Strengthening activity				
Min/week	30 ± 61	30 ± 61	29 ± 58	0.21
MET-minutes/week	118 ± 243	118 ± 243	117 ± 234	0.21
Leisure-time PA				
Min/week	241 ± 243	243 ± 244	193 ± 222	< 0.0001
MET-minutes/week	1046 ± 1060	1056 ± 1064	848 ± 955	< 0.0001
Household PA				
Min/week	381 ± 470	387 ± 474	265 ± 367	< 0.0001
MET-minutes/week	1494 ± 1903	1520 ± 1920	1005 ± 1462	< 0.0001
Outdoor PA				
Min/week	319 ± 379	325 ± 382	208 ± 307	< 0.0001
MET-minutes/week	1327 ± 1614	1351 ± 1626	861 ± 1279	< 0.0001
Indoor PA				
Min/week	298 ± 312	302 ± 315	232 ± 255	< 0.0001
MET-minutes/week	1206 ± 1240	1219 ± 1248	949 ± 1041	< 0.0001
Total walking (*outdoor and walking pet*)				
Min/week	125 ± 190	127 ± 190	89 ± 167	< 0.0001
MET-minutes/week	427 ± 636	433 ± 639	304 ± 560	< 0.0001
Sedentary time				
Min/day	434 ± 205	432 ± 204	470 ± 216	< 0.0001

*P values from Wilcoxon test for unadjusted associations between domains and types of PA and ELISA-S test status.

### Association of PA domains and types with SARS-CoV-2 infection

**Figure 2:**
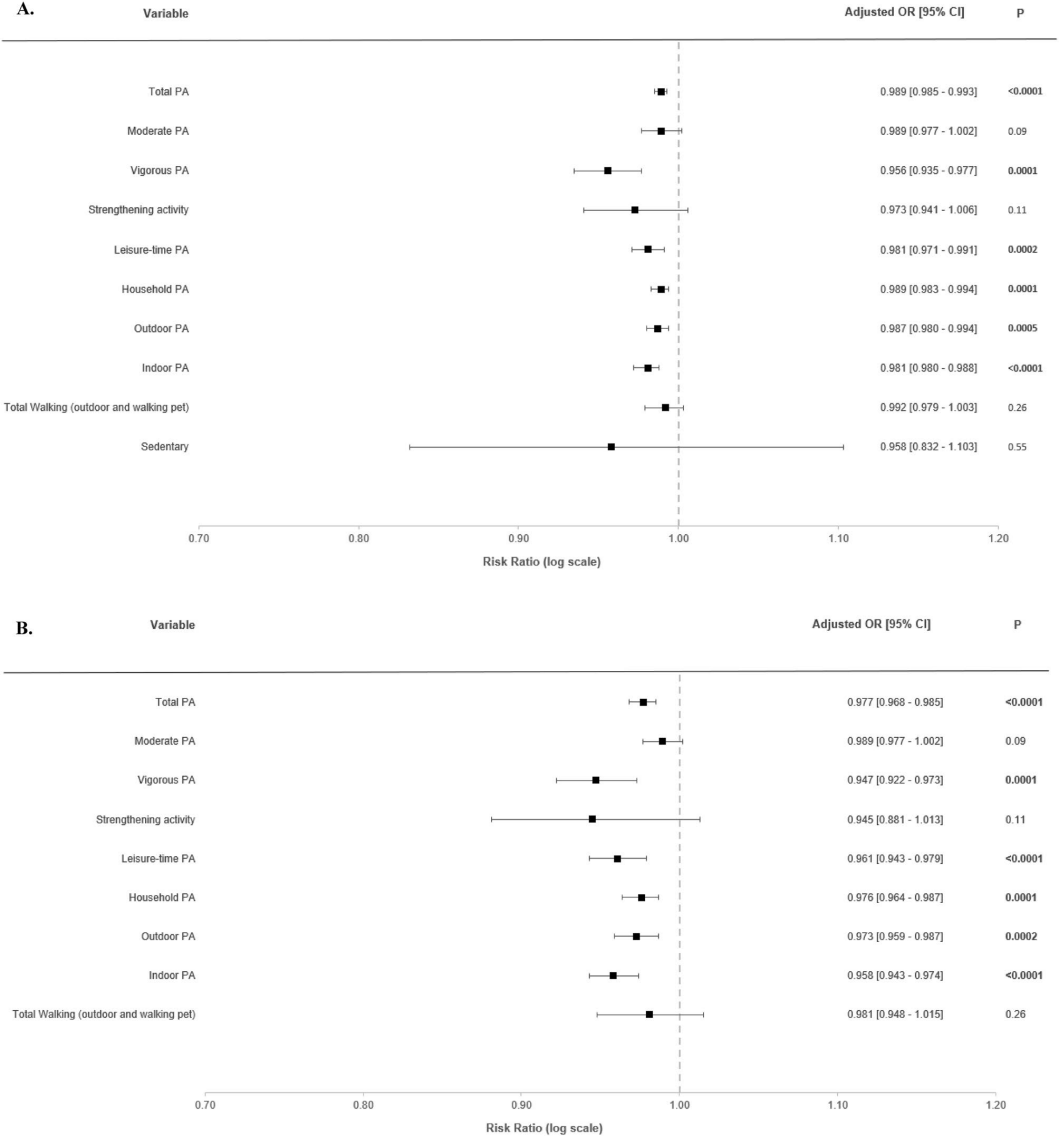
Associations between physical activity and risk of SARS-CoV-2 infection (ELISA-S), NutriNet-Santé cohort, 2020—SAPRIS-SERO, France. ELISA-S positive (n = 1091) compared to ELISA-S negative (n = 21,074) participants. Odds ratios and 95% confidence intervals per 30-min/week increments (**A)** and 250 MET-minutes/week increment (**B**) were obtained from multi-adjusted logistic regression models including sex (men/women), age, educational level (< high-school degree/high-school degree/undergraduate degree/graduate degree), employment status (no professional activity prior to lockdown: unemployed, retired, homemaker/short-time working/working outside home/working from home/student, trainee and other), smoking status (non-smoker, former smoker, smoker), presence of children and/or grandchildren aged under 18 years at home (yes/no), residential area (rural area/city < 20,000 inhabitants/city ≥ 20,000 to 100,000 inhabitants/city > 100,000 inhabitants), frequency of going out over the past week (never/once/2 to 5 times/6 to 10 times/ > 10 times), presence of chronic disease (yes/no), geographical area (Paris Basin/Centre-East/East/Mediterranean/North/West/Paris region/Southwest), BMI, month of blood draw (May–June/July/August–September–October), close relatives with COVID-19 symptoms (yes/no), and a composite score reflecting the adherence to recommended protective behaviors (range 0 to 9).

Associations of PA domains and types with the odds of SARS-CoV-2 infection from multi-adjusted logistic regression models are shown on Fig. 2. Odds are expressed for an increase of 30 min per week (Fig. 2A) and 250 MET-minutes/week (Fig. 2B). Each 30-min/week increment increase in total PA (OR = 0.989; 95% CI 0.985–0.993; *p* < 0.0001), vigorous PA (OR = 0.956; 95% CI 0.935–0.977; *p* < 0.0001), leisure-time PA (OR = 0.981; 95% CI 0.971–0.991; *p* = 0.0002), household PA (OR = 0.989; 95% CI 0.983–0.994; *p* < 0.0001), outdoor PA (OR = 0.997; 95% CI 0.980–0.994; *p* = 0.0005) and indoor PA (OR = 0.981; 95% CI 0.972–0.988; *p* < 0.0001) were all associated with lower odds of SARS-CoV-2 infection (Fig. 2A). No association was found for moderate PA, strengthening activity, total walking and sedentary time with the risk of SARS-CoV-2 infection (Fig. 2A). Similar results were found when PA data were expressed in MET-minutes/week (Fig. 2B).

Secondary analyses with additional adjustment are displayed in Supplementary Files (Figs. S1 and S2). Similar results were found when further adjusting for daily pre-lockdown PA assessed by the IPAQ (Fig. S1). When analyses were adjusted for energy intake (without alcohol), moderate PA (OR = 0.96; 95% CI 0.92–1.00; *p* < 0.05) became associated with lower odds of SARS-CoV-2 infection (Fig. S2).

Sensitivity analyses on the nested case–control sample are also presented in a Supplementary File (Fig. S3). Similar results were found, i.e. total PA, vigorous aerobic PA, leisure-time PA, household PA, outdoor PA and indoor PA were associated with lower odds of SARS-CoV-2 infection.

## Discussion

In this study, we investigated relationships between specific domains and types of PA during lockdown and the risk of testing positive for SARS-CoV-2 infection based on objective seroprevalence data in a large population-based cohort, accounting for many potential confounding factors. Our results indicate an inverse association between not only overall PA but also several PA domains and subtypes (such as leisure-time PA, household PA, outdoor and indoor PA) and the risk of SARS-CoV-2 infection. These data reinforce the importance of PA promotion and wide dissemination of PA guidelines to citizens as part of general measures for counteracting the spread of COVID-19 and attempting to minimize its adverse consequences. Overall, these findings provide support to the importance of PA at population level to mitigate the effects of acute health crisis such as the COVID-19 pandemic, in addition to the well-established protective effects of PA in the fight against NCDs.

A first result of this study is that time spent in total PA during the first French lockdown was associated with a lower risk of SARS-CoV-2 infection. This finding brings new information compared to previous observations in the literature, which suggested that adults engaged in regular PA prior to the COVID-19 pandemic had a lower risk of SARS-CoV-2 infection^10–13^. For example, in a recent systematic review and meta-analysis, authors reported that performing regular PA was associated with an 11% lower prospective risk of infection by SARS-CoV-2^15^. Similar results were also found for the association between regular PA and infectious diseases and acute respiratory infections^8,9^. Authors concluded that having a higher daily PA leads to a lowered risk of community-acquired infectious diseases and an increase in the strength of the mucosal immune barrier^9^. Therefore, this first result on total PA was rather expected. To continue improving the understanding of the links between PA and risk of infection by SARS-CoV-2 and to be able to draw recommendations, it was important to explore in more detail the PA domains and types that may have potential protective effects.

Results from our study bring new evidence showing that various categories of PA—but not all—may play a role in the reduction of SARS-CoV-2 risk of infection. Of note is the fact that our study is the first to investigate the associations between different PA intensity (e.g. vigorous, moderate), different PA domains (e.g. leisure, household) and different PA types (e.g. indoor, outdoor) during the lockdown and the risk of infection by SARS-CoV-2. Findings obtained with PA intensity and those with PA domains and types provide different views of relations with PA. First, there is major interest in analysing PA by intensity, especially for MVPA. Some studies showed that vigorous PA is beneficial on a number of cardiometabolic health outcomes, independent of moderate PA^26–28^. Our study clearly showed that adults who performed vigorous PA had a lower infection risk whereas associations for moderate PA did not reach statistical significance. Even if an association with moderate PA was found after adjustment for energy intake, it remained tangential. Importantly, most previous studies did not account for PA intensity^10,13^. Only, Cho et al.^12^ investigated PA by intensity with infection risk of SARS-CoV-2 using self-report. Authors reported a significant inverse association for both moderate and vigorous PA^12^. Methodological disparities with our study relating to sample size and adjustment for confounding factors might explain the discrepancies between our results and those from this previous study. Another possible explanation relies on the specific activities classified as of moderate intensity, such as walking. It could be hypothesized that total walking, which we did not find associated with infection risk, mitigates the association with moderate PA. When looking at PA domains and types, we found that higher time spent in leisure-time PA, as well as household, outdoor and indoor PA was associated with a decreased risk of infection. Results were unaltered after additional adjustment for lifestyle habits, such as daily PA levels assessed at least 6 months prior to the pandemic crisis. These findings illustrate the importance to take into account various domains and types, and to assess their specific relationships with the SARS-CoV-2 infection.

Several potential mechanisms could explain our results. There is robust evidence of causative links between PA, improved immunity, and disease prevention. In a review on the link between PA and the body’s defense system, Niemans and Wentz^7^ indicated that regular PA may be considered as an important immune system adjuvant to stimulate the ongoing exchange of distinct and highly active immune cell subtypes between the circulation and tissues. PA enhances immune defence by increased immunoglobulins, anti-inflammatory cytokines, neutrophils, NK cells, cytotoxic T cells, and immature B cells and reduced chronic inflammation^7^. In a previous epidemiological study, authors reported that the number of days with upper respiratory tract infection during the 12-week period was significantly reduced by 43% in subjects engaging in an average of 5 or more days per week of aerobic exercise (20 min bouts or longer) compared to those who were minimally active (≤ 1 day/week)^29^. Single bouts of moderate PA are also beneficial for the immune system and considered as immuno-enhancing^30^. Nevertheless, very intense and prolonged PA seem to have adverse effects on the immune system with a temporary immunodepression leading to an increased risk of infection^7^. This fact is well established in elite athletes^31^. We found that vigorous PA was significantly associated to a low odd of SARS-CoV-2 infection. However, this difference between our results and previous literature may be explained by the population studied and the definition of vigorous PA level. Indeed, the majority of this previous literature is devoted to the acute effect of exercises and focuses only on athletes with highest PA levels. Authors reported that training sessions at high intensity and a high participation to several tournaments in a short term leading a transient immune perturbation in elite athletes^7^. Lastly, engaging in regular PA have a potent anti-inflammatory effect and also reduce the risk of overweight and obesity, strongly associated with SARS-CoV-2 infection^1,32^.

Our study presented several strengths and limitations. First, PA was assessed by a self-reported questionnaire. Thus, classification errors from social desirability, lack of awareness, or perceptual bias could not be ruled out. This may lead to over or underreporting of PA level, and by consequence, may mislead the estimation of the magnitude of studied associations^33^. However, the social desirability bias may be reduced in web-based research compared to the traditional paper-and-pencil method^34^. Second, since participation to the NutriNet-Santé cohort is made on a voluntary basis, individuals tended to be healthier, including more women and with higher education compared to the general French population^35^. Thus, caution is needed in the extrapolation of our results. Next, the cross-sectional observational design of the study does not allow us to draw conclusions in terms of causality of observed relationships. Indeed, our study design does not allow for the examination of the temporal relationship, between physical activity and SARS-Cov2 infection. However, our models included thorough adjustments for a wide range of potential confounders, they are consistent with previous studies on total PA and SARS-CoV-2 infection and are supported by plausible mechanistic interpretation, which are important argument in favor of causality. Last, studies have suggested that the ELISA test has imperfect sensitivity (85–90%) and that anti-SARS-CoV-2 antibodies may decrease over time, which may have resulted in misclassification in a way that a person who has been infected with the virus might have not been tested as positive according to the ELISA test^36,37^. However, the date of collection of dried-blood spots was between May and October 2020 (i.e. close to the beginning of the pandemic in France, and yet, after the PA exposure assessment, for a prospective design). In addition, we were unable to know the date of infection with limits of seroprevalence analyses, and therefore could be prior to PA assessment. The main strength is that the first study with a large sample size of French adults to assess relationships between different PA domains and types and the risk of SARS-CoV-2 infection. Another strength of this study is the inclusion of several confounding factors in the statistical analyses which was not included in previous studies, such as protective barriers behaviours (mask wearing, hand washing, etc.), as well as the possibility to test adjustment for energy intake. Lastly, our study was based on a comprehensive assessment of SARS-CoV-2 seroprevalence with highly sensitive assays, independent of whether or not the participant sought testing (contrary to studies using PCR results retrieved from medical record) or even had symptoms (that might not be specific to SARS-CoV-2 infections).

In conclusion, our results indicate an inverse association between not only overall PA but several PA domains and types and SARS-CoV-2 infection. These findings contribute to an enhanced understanding of the relationships between PA domains and types during the specific lockdown setting and SARS-CoV-2 infection. Along with the already well-established benefits of PA for non-communicable disease prevention, these findings provide additional evidence for policies promoting all types of PA as a lever for improved population health.

### Supplementary Information

Supplementary Figures.

## Data Availability

The datasets used and/or analysed during the current study available from the corresponding author on reasonable request. Researchers from public institutions can submit a collaboration request including their institution and a brief description of the project to collaboration@etude-nutrinet-sante. All requests will be reviewed by the steering committee of the NutriNet-Santé study. A financial contribution may be requested. If the collaboration is accepted, a data access agreement will be necessary and appropriate authorizations from the competent administrative authorities may be needed. In accordance with existing regulations, no personal data will be accessible.
