# Supplementary material for: Association of SARS-CoV-2 infection with physical activity domains and types
Source: Sci Rep. 2023 Nov 6;13:19187. doi: 10.1038/s41598-023-46162-4 (PMC10628273; doi:10.1038/s41598-023-46162-4)
Supplement: Supplementary file 1 — Supplementary Figures. [file 41598_2023_46162_MOESM1_ESM.pdf]

A.

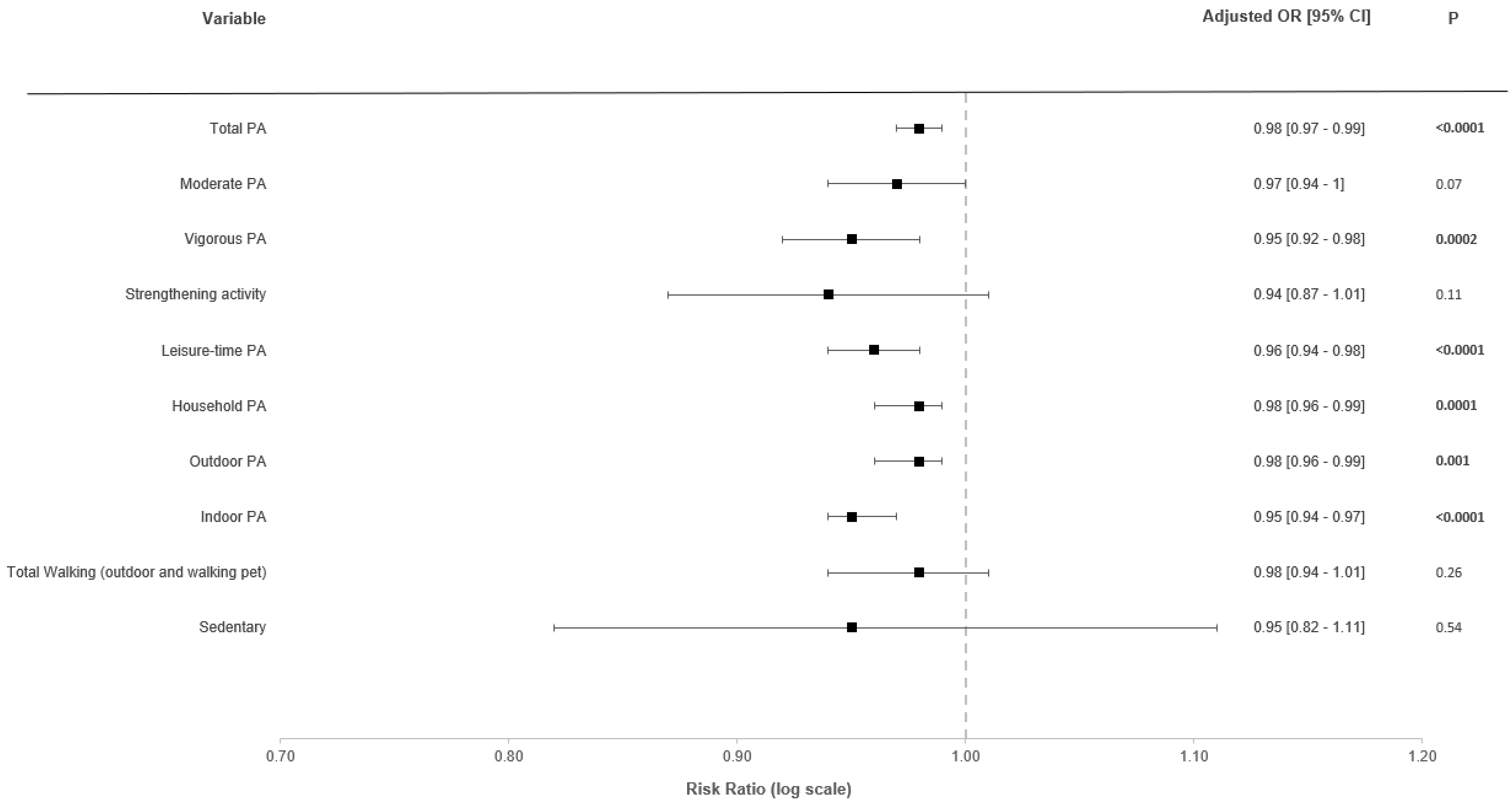
Variable
Adjusted OR [95% CI]
P
Total PA
0.98 [0.97 - 0.99]
<0.0001
Moderate PA
0.97 [0.94 - 1]
0.07
Vigorous PA
0.95 [0.92 - 0.98]
0.0002
Strengthening activity
0.94 [0.87 - 1.01]
0.11
Leisure-time PA
0.96 [0.94 - 0.98]
<0.0001
Household PA
0.98 [0.96 - 0.99]
0.0001
Outdoor PA
0.98 [0.96 - 0.99]
0.001
Indoor PA
0.95 [0.94 - 0.97]
<0.0001
Total Walking (outdoor and walking pet)
0.98 [0.94 - 1.01]
0.26
Sedentary
0.95 [0.82 - 1.11]
0.54
0.70
0.80
0.90
1.00
1.10
1.20
Risk Ratio (log scale)

B.

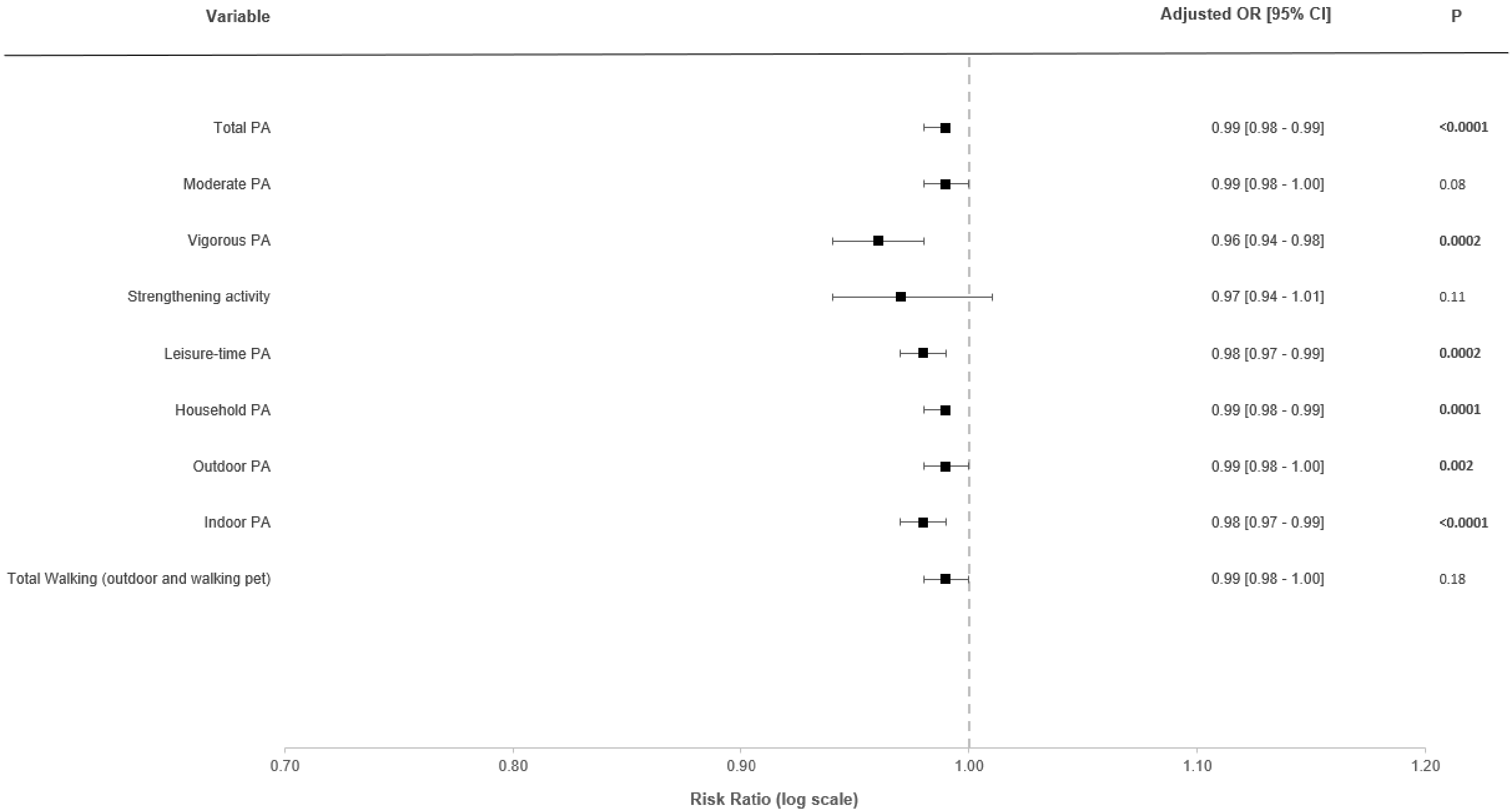

Supplementary Figure S1. Association between physical activity and risk of SARS-CoV-2 infection (ELISA-S), Nutrinet-Santé cohort (2009-2020) – SAPRIS-SERO, France. ELISA-S positive (n=999) compared to ELISA-S negative (n=19887) participants. Odds ratios and 95% confidence intervals per 30-min/week increments for PA and 420-min/week for sedentary time (**A**) and 250 MET-minutes/week increment (**B**) were obtained from multi-adjusted logistic regression models including sex (men/women), age, educational level (< high-school degree/high-school degree/undergraduate degree/graduate degree), employment status (no professional activity prior to lockdown: unemployed, retired, homemaker/short-time working/working outside home/working from home/student, trainee and other), smoking status (non-smoker, former smoker, smoker), presence of children and/or grandchildren aged under 18 years at home (yes/no), residential area (rural area/city < 20,000 inhabitants/city ≥ 20,000 to 100,000 inhabitants/city > 100,000 inhabitants), frequency of going out over the past week (never/once/2 to 5 times/6 to 10 times/> 10 times), presence of chronic disease (yes/no), geographical area (Paris Basin/Centre-East/East/Mediterranean/North/West/Paris region/Southwest), BMI, month of blood draw (May–June/July/August-September–October), close relatives with COVID-19 symptoms (yes/no), a composite score reflecting the adherence to recommended protective behaviors (range 0 to 9) and daily PA levels before the lockdown assessed by IPAQ (low, moderate, high).

A.

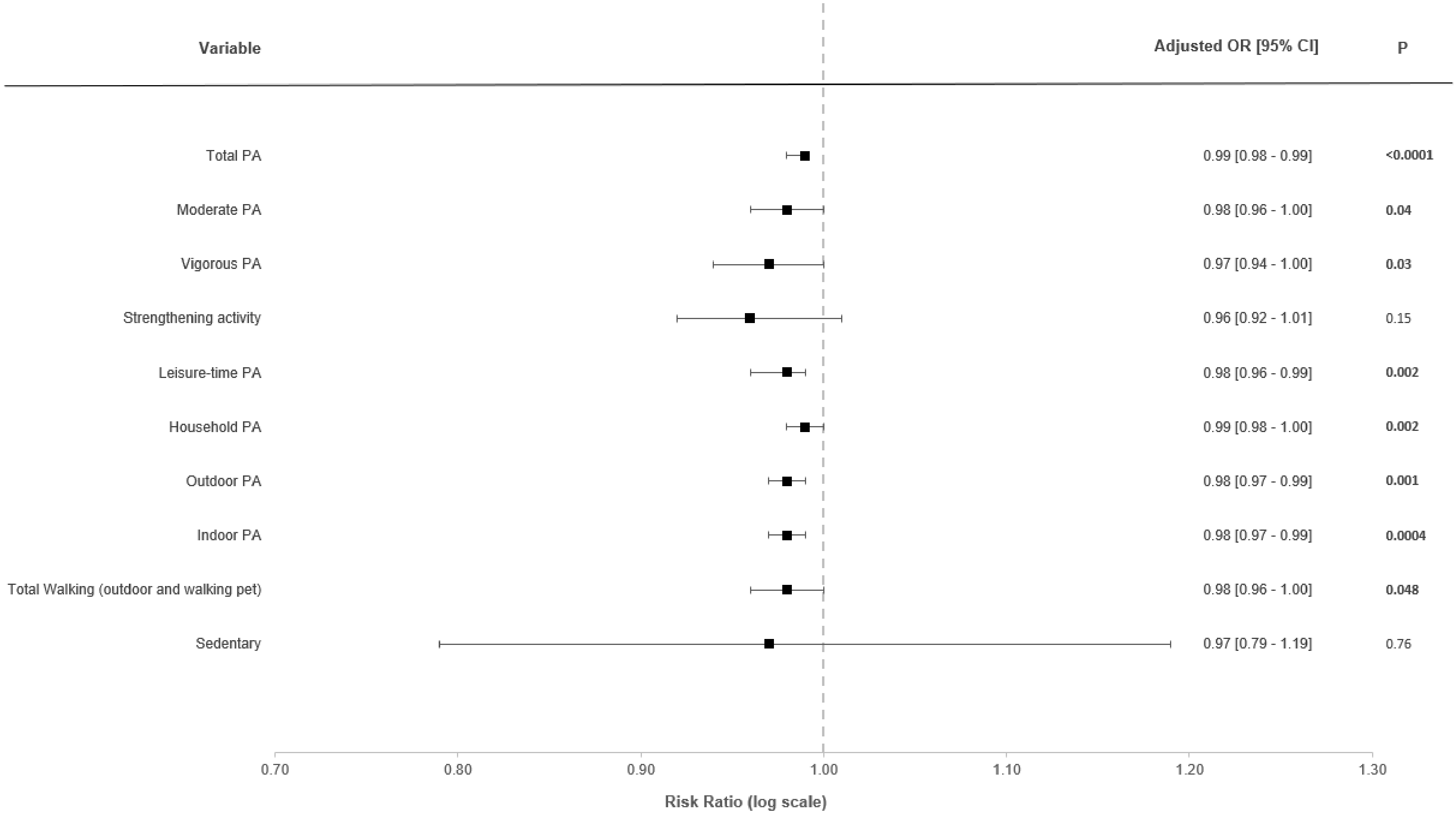
Variable
Adjusted OR [95% CI]
P
Total PA
0.99 [0.98 - 0.99]
<0.0001
Moderate PA
0.98 [0.96 - 1.00]
0.04
Vigorous PA
0.97 [0.94 - 1.00]
0.03
Strengthening activity
0.96 [0.92 - 1.01]
0.15
Leisure-time PA
0.98 [0.96 - 0.99]
0.002
Household PA
0.99 [0.98 - 1.00]
0.002
Outdoor PA
0.98 [0.97 - 0.99]
0.001
Indoor PA
0.98 [0.97 - 0.99]
0.0004
Total Walking (outdoor and walking pet)
0.98 [0.96 - 1.00]
0.048
Sedentary
0.97 [0.79 - 1.19]
0.76
0.70
0.80
0.90
1.00
1.10
1.20
1.30
Risk Ratio (log scale)

B.

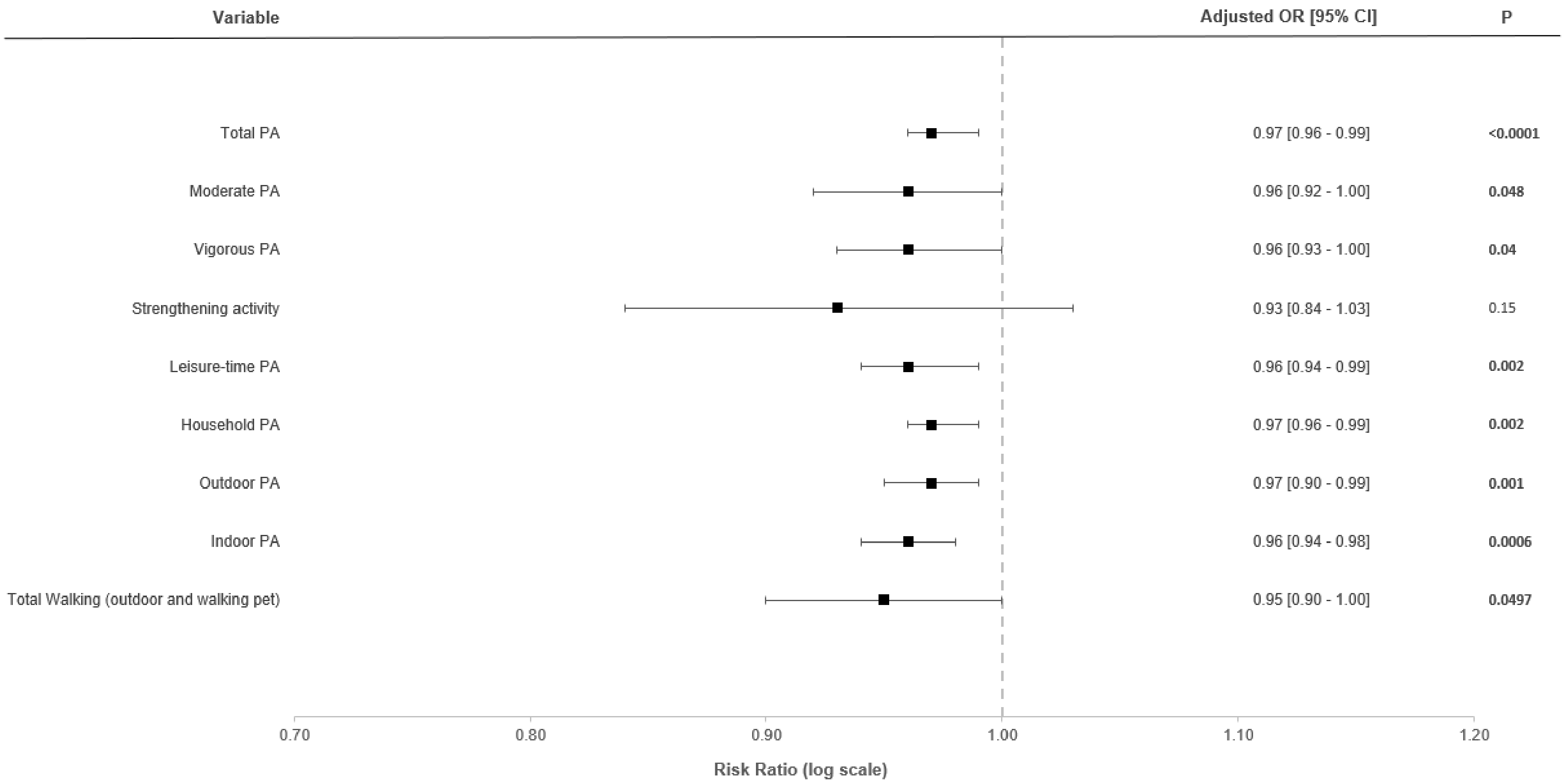

Supplementary Figure S2. Association between physical activity and risk of SARS-CoV-2 infection (ELISA-S), Nutrinet-Santé cohort (2009-2020) – SAPRIS-SERO, France. ELISA-S positive (n=548) compared to ELISA-S negative (n=11755) participants. Odds ratios and 95% confidence intervals per 30-min increments for PA and 420-min/week for sedentary time (**A**) and 250 MET-minutes increment (**B**) were obtained from multi-adjusted logistic regression models including sex (men/women), age, educational level (< high-school degree/high-school degree/undergraduate degree/graduate degree), employment status (no professional activity prior to lockdown: unemployed, retired, homemaker/short-time working/working outside home/working from home/student, trainee and other), smoking status (non-smoker, former smoker, smoker), presence of children and/or grandchildren aged under 18 years at home (yes/no), residential area (rural area/city < 20,000 inhabitants/city ≥ 20,000 to 100,000 inhabitants/city > 100,000 inhabitants), frequency of going out over the past week (never/once/2 to 5 times/6 to 10 times/> 10 times), presence of chronic disease (yes/no), geographical area (Paris Basin/Centre-East/East/Mediterranean/North/West/Paris region/Southwest), BMI, month of blood draw (May–June/July/August-September–October), close relatives with COVID-19 symptoms (yes/no), a composite score reflecting the adherence to recommended protective behaviors (range 0 to 9) and energy intakes (without alcohol, kcal/day) assessed by 3 nonconsecutive 24-hour dietary records, after exclusion of under-reporters.

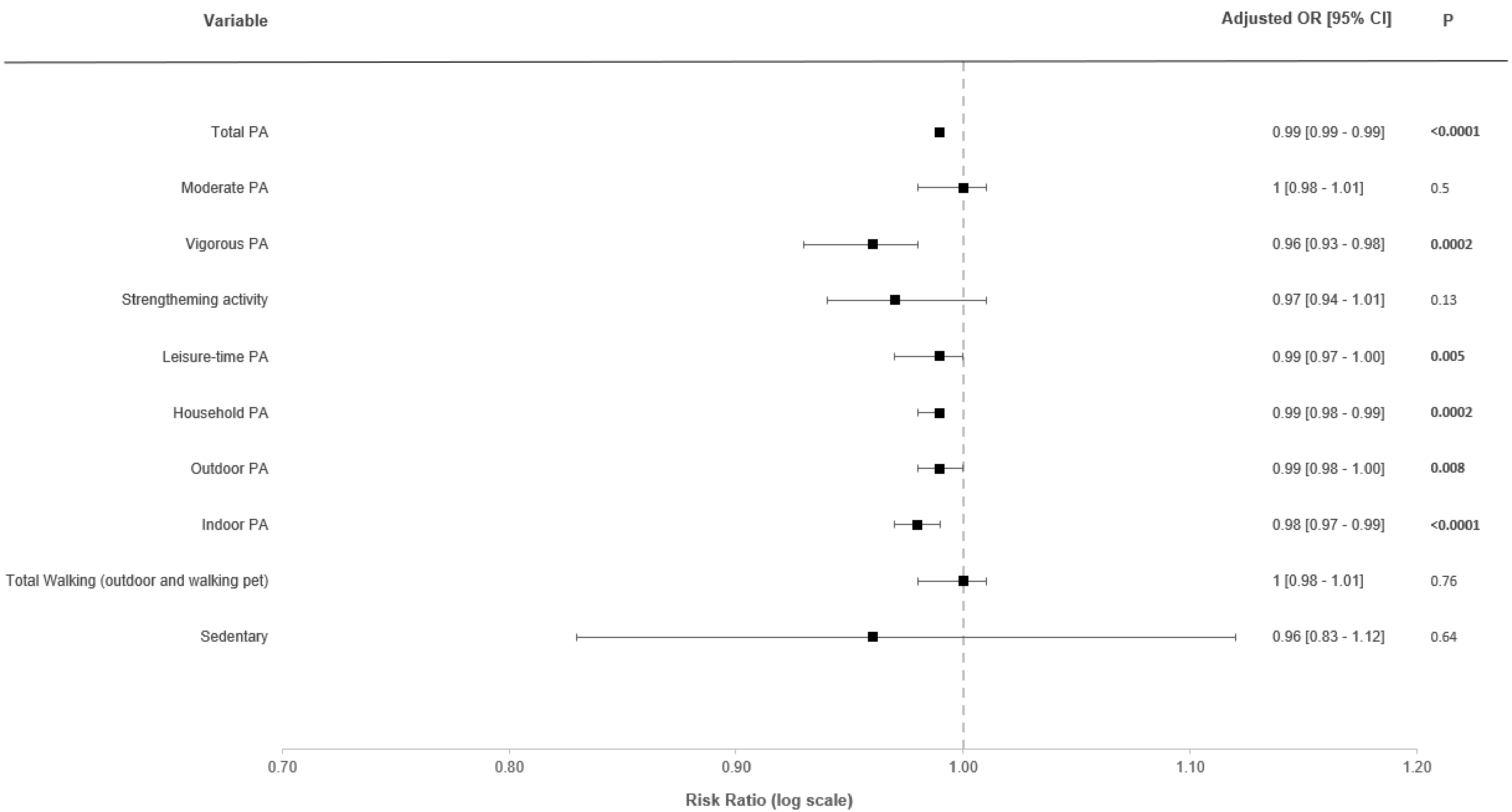

Supplementary Figure S3. Association between physical activity and risk of SARS-CoV-2 infection (ELISA-S), nested case-control design, NutriNet-Santé cohort (2009-2020) – SAPRIS-SERO, France. ELISA-S positive (n=1091) compared to ELISA-S negative (n=4364) controls, matched for age, sex and residential area. Odds ratios and 95% confidence intervals per 30 min increment for PA and 420-min/week for sedentary time obtained from multi-adjusted logistic regression models including sex (men/women), age, educational level (< high-school degree/high-school degree/undergraduate degree/graduate degree), employment status (no professional activity prior to lockdown: unemployed, retired, homemaker/short-time working/working outside home/working from home/student, trainee and other), smoking status (non-smoker, former smoker, smoker), presence of children and/or grandchildren aged under 18 years at home (yes/no), residential area (rural area/city < 20,000 inhabitants/city ≥ 20,000 to 100,000 inhabitants/city > 100,000 inhabitants), frequency of going out over the past week (never/once/2 to 5 times/6 to 10 times/> 10 times), presence of chronic disease (yes/no), geographical area (Paris Basin/Centre-East/East/Mediterranean/North/West/Paris region/Southwest), BMI, month of blood draw (May–June/July/August-September–October), close relatives with COVID-19 symptoms (yes/no) and a composite score reflecting the adherence to recommended protective behaviors (range 0 to 9).
